# Distress reactions and susceptibility to misinformation for an analogue trauma event

**DOI:** 10.1186/s41235-024-00582-6

**Published:** 2024-08-26

**Authors:** Prerika R. Sharma, Emily R. Spearing, Kimberley A. Wade, Laura Jobson

**Affiliations:** 1 Turner Institute for Brain and Mental Health and School of Psychological Sciences, Monash University, Melbourne, Australia; 2 Law School, University of Exeter, Exeter, UK; 3 Department of Psychology, University of Warwick, Coventry, UK

**Keywords:** Distress, Misinformation, False memories, Traumatic memory, Avoidance

## Abstract

Accuracy of memory is critical in legal and clinical contexts. These contexts are often linked with high levels of emotional distress and social sources that can provide potentially distorting information about stressful events. This study investigated how distress was associated with susceptibility to misinformation about a trauma analogue event. We employed an experimental design whereby in Phase 1, participants (*N* = 243, aged 20–72, 122 females, 117 males, 4 gender diverse) watched a trauma film (car crash) and heard an audio summary that contained misinformation (misled items), true reminders (consistent items), and no reminders (control items) about the film. Participants rated their total distress, and symptoms of avoidance, intrusions, and hyperarousal, in response to the film. They then completed cued recall, recognition, and source memory tasks. One week later in Phase 2, participants (*N* = 199) completed the same measures again. Generalised linear mixed models were used. A significant misinformation effect was found, and importantly, participants with higher distress levels showed a smaller misinformation effect, owing to especially poor memory for consistent items compared to their less distressed counterparts. Distress was also associated with improved source memory for misled items. Avoidance of the film’s reminders was associated with a smaller misinformation effect during immediate retrieval and a larger misinformation effect during delayed retrieval. Findings suggest that distress is associated with decreased susceptibility to misinformation in some cases, but also associated with poorer memory accuracy in general. Limitations are discussed, and the need for further research is highlighted.

**Supplementary Information:**

The online version contains supplementary material available at 10.1186/s41235-024-00582-6.

## Significance statement

In the legal and clinical systems, individuals’ memories inform important forensic decision-making and therapeutic decisions, respectively. Individuals often interact with these contexts to report or process past distressing events, which involves discussion with others such as co-witnesses and therapists. It is possible that others may misremember an event and unknowingly share incorrect details about past events. The *misinformation effect* shows how our memory of an event can be distorted by misleading suggestions we have gleaned from others. But how susceptible are distressed individuals to such misinformation? The current study investigated this. 243 participants watched a film about a car crash, rated their distress, received verbal misinformation about the film, and completed three different memory tests. 199 participants returned for the same memory tests one week later. We inspected participants’ memory of the film’s details they were misled about (misled items), and true details they did (consistent items) and did not (control items) receive reminders about. A strong misinformation effect was found. Greater distress was associated with a smaller misinformation effect, owing to poorer memory for consistent items. Participants who tried to avoid reminders of the film showed less susceptibility to misinformation during immediate testing but greater susceptibility in delayed testing. Distressed participants showed better ability to correctly remember the source of the misinformation. These results suggest that distress is associated with reduced susceptibility to misinformation in some cases but also with poorer memory accuracy. Limitations and the need for further research are discussed.

## Introduction

Ensuring accuracy of memory processes is desirable in most contexts but critical in some. The legal system operates on the expectation that individuals can recall and report accurate memories, often about stressful events. In clinical settings, treatment-seeking individuals often recall past traumatic events while trying to make sense of these memories (see Wright, 2020). Inaccurate memories within these contexts can have serious implications, by impacting forensic decisions and affecting individuals’ psychological well-being, respectively. Additionally, in these contexts, individuals may often experience high levels of distress at various stages, such as during encoding and retrieval of a memory. Research shows that memory is impacted by several factors, with individuals’ emotional states being a key influence (e.g. Deffenbacher et al., 2004; Kensinger, 2009). This influence does not always ensure veracity of our memories, and memory distortions are common even for emotional events (Bookbinder & Brainerd, 2016). For example, we may recall negative events particularly well but may also be prone to incorporating inaccurate information within these memories, as keeping such memories updated is important to our survival (Porter et al., 2008). Our memory is also impacted by factors external to us. We often engage in conversations about mutually witnessed or experienced events with others such as friends or family, and information gathered from such social sources can impact the way we remember events (see Loftus, 2005; Wessel & Moulds, 2008). Certainly, in the legal and clinical settings, many social sources exist, such as co-witnesses and therapists. Information gleaned from these sources is not always accurate; individuals may unknowingly share erroneous information—or misinformation—about events through their own accounts of experiences or through suggestive questioning styles. The rememberer may incorporate these inaccurate suggestions within their own memory of the event, resulting in suggestion-induced memory distortions (Otgaar et al., 2017). Given the emotional intensity that is associated with these contexts, how likely are individuals to accept these suggestions? How does their distress impact how susceptible they may be to suggestions? The current study sought to understand this. Specifically, we investigated how levels of distress were associated with the development of suggestion-induced memory distortions for traumatic material: an under-researched topic.

In the trauma literature, distress reactions are conceptualised as avoidance (voluntary efforts to avoid trauma-related reminders), intrusions (involuntary reminders of the traumatic event), and hyper- or hypo-arousal (symptoms of excess or reduced physiological arousal) (Clapp et al., 2020; Creamer et al., 2003). To understand the effects of distress on memory distortion, it is helpful to first consider how stress affects memory. Theoretically, it has been posited that stress leads to a shift towards a rigid, habitual memory system, which allows for efficient processing and enhanced consolidation of stressful events (see review by Quaedflieg & Schwabe, 2018). At the same time, this shift in focus entails reduced memory specificity, reduced incorporation of contextual details associated with such memories, and prevents memories from being updated with new information (Quaedflieg & Schwabe, 2018). In this way, the experience of stress can prevent the incorporation of misinformation into the memory of the event (Quaedflieg & Schwabe, 2018). Although this shift is helpful when experiencing the stressful event, continued use of these systems past the stressful event can negatively affect memory by leaving knowledge unintegrated and inflexible (Conway, 2005; Ehlers & Clark, 2000; Quaedflieg & Schwabe, 2018). Thus, over time, stress may lead to gaps in knowledge that may be filled with other information, such as suggestions from others, leading to greater incorporation of misinformation (Quaedflieg & Schwabe, 2018).

The experience of psychological distress is prominent in the relationship between stress and memory. Avoidance and intrusions can repeatedly activate the representations of the stressful content and prolong its negative impact on memory (Brosschot et al., 2005; Korten et al., 2014). Avoidance and intrusions have been found to predict the reduction in autobiographical memory specificity that is associated with trauma exposure (Geraerts et al., 2010; Stephens et al., 2013 but see Phung & Bryant, 2013; Wessel et al., 2002) and have also been associated with poorer working memory performance (Korten et al., 2014). At the same time, negative and traumatic memories are associated with heightened subjective recall (e.g. increased vividness, Blix et al., 2020; Peace et al., 2008) and emotional intensity (Rubin et al., 2008) but may be subject to inconsistencies in accuracy over time (Giosan et al., 2009; Engelhard et al., 2008 but see Alexander et al., 2005). Thus, distress can have significant negative impacts on memory processes. What role does distress play in distorting memories?

Suggestion-induced memory distortions are commonly measured using the misinformation paradigm (Loftus, 1992; Loftus & Hoffman, 1989), whereby participants encode an event, unknowingly receive misinformation about the event, and complete a memory test. The misinformation effect describes the robust finding that participants often incorrectly endorse the suggested, misleading detail as belonging to the originally viewed material. There are limited studies investigating the relationship between distress and the misinformation effect, with a recent review finding only three relevant studies (Sharma et al., 2022). Avoidance or suppression strategies have commonly been measured in studies. Consistent with the impacts of stress on memory (Quaedflieg & Schwabe, 2018), short-term use of avoidance has been found to protect against misinformation (Monds et al., 2013; Moore & Zoellner, 2012 but see Monds et al., 2016). Another account of this finding is provided by the cognitive load model (Lavie et al., 2004; Moore & Zoellner, 2012), which postulates that suppression, being a cognitively effortful task, may broaden attention leading to irrelevant details being encoded rather than the task at hand. Attention may be moved away from misinformation being received and towards other contextual details not immediately related to the film (Moore & Zoellner, 2012), thus reducing suggestion-induced distortions. Additionally, immediate intrusions have been associated with greater recall (Monds et al., 2013) and recognition (Monds et al., 2016) accuracy, which may be due to repeated rehearsal of the event (Monds et al., 2013). Similar to avoidance, intrusions also act as repetitive thinking about the stressful experience and can prolong the negative representations of stress (Korten et al., 2014). Thus, similar to avoidance, one would expect intrusions to protect against misinformation in the short term. However, within the limited studies, intrusions have not been associated with susceptibility to misinformation (Monds et al., 2013, 2016).

As time passes, one would expect susceptibility to misinformation to increase (Loftus, 2005) and the continued use of the stress-induced habitual memory system to impair memory (Quaedflieg & Schwabe, 2018). However, findings on the effect of persistent distress are inconsistent. Avoidance of trauma film-related material that persists for 48 h post-encoding may guard against misinformation on a recognition test (Moore & Zoellner, 2012). Avoidance persisting for one week instead may increase misinformation on a free recall test (Monds et al., 2013), although this was not found by Monds et al. (2016) using a similar design. When negative photographs instead of films were used, even day-long distress has been associated with greater susceptibility to misinformation (Nahleen et al., 2022). However, this study used static stimuli (i.e. photographs), which are less memorable (Candan et al., 2015) and ecologically valid than dynamic stimuli (i.e. film footage). Thus, within these limited studies, the materials, designs, and conclusions vary widely. The inconsistent pattern of results may reflect differences in the type of memory test (recall, recognition, and old/new judgements), the type of distress measured (total, avoidance, and intrusions), duration of distress (ranging from immediate to one week), or gender differences (as Moore & Zoellner used a woman-only sample). Additionally, none of these studies measured source memory, which refers to memory of the source where a detail was encountered (e.g. hearing a detail from your friend versus from a video; Lindsay & Johnson, 1989). Source memory can provide important information about suggestion-induced memory distortions, such as providing some evidence as to whether the misleading information may have ‘updated’ the original memory or the distortion reflects a confusion in selecting between two sources. Finally, none of the above studies included hyperarousal as a measure of distress, despite its importance in understanding distress and trauma severity (Clapp et al., 2020). Given the important implications of distress on suggestion-induced memory distortions, further research in this area is needed.

The aim of this study was to investigate how distress affected the misinformation effect across three types of memory test: cued recall, recognition, and source memory. We asked participants to view a distressing film about a car crash and then gave them (a) verbal misleading details (misled items), (b) true and repeated details (consistent items), or (c) no repeated details (control items) about various critical details in the film. We then tested their memory of the film using three memory tests both immediately and one week later. The first aim of this study was to assess whether memory for a trauma analogue event is prone to misinformation. Although it is now recognised that traumatic events are prone to distortion (Lommen et al., 2013; Strange & Takarangi, 2015), the robustness of the misinformation effect for traumatic material is under-researched (Sharma et al., 2022). We hypothesised that accuracy would be significantly lower on misled items than on control and consistent items (i.e. the misinformation effect: Hypothesis 1). The second aim was twofold: to investigate how distress reactions to a trauma analogue event were associated with susceptibility to misinformation and how these associations changed over time. Based on the effects of stress on memory over time (Quaedflieg & Schwabe, 2018), we hypothesised that at immediate retrieval, higher levels of distress would be significantly associated with greater accuracy on misled items (i.e. reduced misinformation effect: Hypothesis 2a), and at one-week-delayed retrieval, higher levels of distress over the past week would be significantly associated with lower accuracy on misled items (i.e. increased misinformation effect: Hypothesis 2b).

## Method

### Design

All procedures were approved by the Monash University Human Research Ethics Committee (#31690). The study was preregistered with AsPredicted: https://aspredicted.org/88T_47W (#106038). Our aims were examined using the trauma film paradigm (Holmes et al., 2004; Taylor et al., 2021) and misinformation paradigm (Loftus & Hoffman, 1989). We investigated whether the relationship between item type (3 levels: misled, control, and consistent), retrieval time (2 levels: immediate and 1 week delayed), and self-reported distress (continuous: reported at each retrieval phase) was associated with the probability of being accurate on memory tests. Memory was assessed using cued recall, recognition, and source memory tests immediately following the film (Phase 1) and one week later (Phase 2). We have reported all measures, conditions, data exclusions, and how sample size was determined, and all original study materials, data, and R code are available on Open Science Framework: https://osf.io/6xvzn/?view_only=505386c567be4626a6fbfe8c13aff3c9.

### Participants

We based our power calculations on previous similar studies (Moore & Zoellner, 2012, *n* = 97; Monds et al., 2016, *n* = 105; Nahleen et al., 2020, *n* = 165) and aimed for a larger sample size of 200 participants. As we expected 30% attrition and exclusion of participants due to failed attention check questions or technical difficulties, we aimed to collect data from at least 230 participants in the first phase. To ensure sufficient power, two G*power calculations were performed to estimate minimum sample sizes to detect the three-way interaction (item type × distress × retrieval time). The first calculation with ANOVA repeated measures, within–between interactions with a medium effect size (*f* = 0.25; Moore & Zoellner, 2012), 80% power, and alpha criterion of 0.05 yielded a minimum sample size of 42 participants. Another calculation with linear multiple regression: *R*^2^ deviation from zero (with predictors including the three-way interaction, two-way interactions, and main effects) with a medium effect size (*f*^2^ = 0.15), 80% power, and alpha criterion of 0.05 yielded a minimum sample size of 103 participants. Thus, the recruitment of 230 participants was expected to sufficiently power the study.

We used the online TurkPrime platform to recruit participants from Amazon’s Mechanical Turk. Participants were eligible if they: (a) were aged between 18 and 65 years, (b) had normal or corrected-to-normal vision, and (c) were not colour-blind. Participants were paid 6 USD for completion of the study.^1^ Post-recruitment exclusion criteria were applied (all preregistered). Participants were excluded if they: (a) reported having watched the film before (*n* = 20), (b) gave incorrect answers on one of the two attention check questions (*n* = 15), (c) completed either of the study phases in multiple sittings (*n* = 2), (d) reported technical difficulties while watching the film or hearing the narrative (*n* = 20), (e) reported pausing or skipping the video (*n* = 4), or f) withdrew participation before completing the entirety of at least one memory test (*n*_phase 1_ = 62 and *n*_phase 2_ = 5). A final exclusion criterion was added once data collection had begun, as data showed presence of a few bot or nonsensical responses; participants were also excluded if g) they responded with non-compliant responses to more than 50% (i.e. 9 questions) of the cued recall questions (*n* = 3).

The final sample comprised 243 participants for Phase 1 (aged 20–72 years, *M* = 40.15 years, *SD* = 10.97), including 122 females, 117 males, 2 non-binary/non-conforming persons, and 2 people who did not disclose their gender. Of these, 201 participants began Phase 2 but only 199 participants completed Phase 2. Thus, 199 participants completed the study in full.

### Materials

#### Film

We used the trauma film paradigm (Holmes et al., 2004). Participants were shown a short film about a car crash (see ‘traumatic film variant’ used in Taylor et al., 2021), wherein three young women are suddenly involved in two car crashes leading to the deaths of several people. The film involved some confronting details, such as a passenger audibly breaking her neck and an unresponsive baby. This film has been used in previous studies for similar purposes of eliciting trauma analogue reactions (Segovia et al., 2017; Strange & Takarangi, 2012)^2^ and has been shown to induce negative affect (Taylor et al., 2021). The film was 2 min and 47 s long. Participants were given the following instructions: ‘Please watch the following film as if you were a bystander witnessing this event in real life. Pay close attention to the details, as you will be asked questions about it later.’ A screen with text instructing the participants to select the ‘full screen’ button and wait for the video to begin was added before the film for seven seconds.

#### Misinformation narratives

We selected 18 objects/aspects to serve as critical items in the film. These were informed by previous studies using the trauma film paradigm (Monds et al., 2013; Segovia et al., 2017) and discussion between authors about the salient and non-salient aspects of the film. Pilot testing informed the final wording and number of questions.^3^ Three misinformation narratives were created, each containing all 18 critical items and describing the film’s events chronologically (293, 301, and 306 words, respectively). The narratives were introduced to the participants as a ‘summary’ of the film. Each narrative contained 6 misled, 6 consistent, and 6 control items, and the critical items were counterbalanced such that, across the three narratives, each item appeared once as a misled item, once as a consistent item, and once as a control item. Misled items included inaccurate post-event information. Consistent items repeated true details from the film. Control items did not repeat any film details. For example, for the critical item enquiring about the cause of death of the passenger who died by breaking her neck, one narrative stated that she hit the dashboard (misled condition), one narrative stated that she broke her neck (consistent condition), while the other narrative did not mention the cause of her death (control condition). The narratives were delivered to participants in the form of an audio recording narrated by a female voice (author PS), without any accompanying written text.

#### Memory tests

Participants completed three different memory tests: cued recall, recognition, and source memory. The cued recall test included 18 open-ended text questions, such as ‘In the first car, how did the woman in the passenger seat die?’ Recognition tests included 18 true-or-false questions, such as ‘The woman in the passenger seat died from smashing her head into the dashboard.’ Finally, in the source memory test, participants were shown 36 details (misleading and consistent details for each of the 18 items) and asked to identify where they encountered each detail: ‘video only,’ ‘summary only,’ ‘video and summary,’ ‘no memory,’ or ‘don’t know.’

#### Positive and negative affect schedule (PANAS)

As commonly used in trauma analogue studies (e.g. Moore & Zoellner, 2012; Nahleen et al., 2020), the PANAS was used to ensure the film induced appropriate affect in participants. The PANAS is a 20-item list of 10 positive and 10 negative affective states that a person may be presently experiencing (Watson et al., 1988). It can reliably measure changes in affect (Haney et al., 2023) and correlates with measures of distress and psychopathology (see Watson et al., 1988). Participants completed the PANAS before and after the film. Scores were only used to measure the effect of the film on participants’ internal states and have not been analysed further.

#### Impact of events scale-revised (IES-R)

The IES-R (Weiss, 2007) is a 22-item self-reported questionnaire that measures traumatic stress reactions in response to a specific event over the past seven days. The IES-R is designed to measure the core phenomena of traumatic stress reactions: intrusions, avoidance, and hyperarousal. The IES-R has been used in previous studies examining distress associated with the trauma film paradigm (Monds et al., 2013; Segovia et al., 2017) and shows good internal consistency (Creamer et al., 2003). For the study’s purposes, participants reported their distress reactions to the film on two occasions: immediately after watching the film and one week later. For the immediate completion, we followed the approach of Monds et al. (2013) and adjusted the IES-R by removing four questions that were inconsistent with the experimental setting (e.g. item 20—‘I had dreams about it’). The final scale comprised 18 items (McDonald’s omega Phase 1_*ω*_: 0.94, Phase 2_*ω*_: 0.94), with the intrusions subscale including 6 items (Phase 1_*ω*_: 0.93, Phase 2_*ω*_: 0.94), avoidance subscale 7 items (Phase 1_*ω*_: 0.83, Phase 2_*ω*_: 0.85), and hyperarousal subscale 5 items (Phase 1_*ω*_: 0.83, Phase 2_*ω*_: 0.92). For comparability, this adjusted IES-R was also used in the delayed retrieval phase. As advised by authors (Weiss, 2007; Weiss & Marmar, 1997 in Creamer et al., 2003), scores were computed as mean scores of the subscales (ranging 0–4) and a total scale score (ranging 0–12).

### Procedure

All participants completed the study online. The purpose of the study was not disclosed to participants; rather, participants were informed that the study investigated the relationship between types of media, attention, and mental health. To help reduce the likelihood of anticipatory stress, participants were informed that they would be randomly allocated to watch an upsetting, frustrating, inspiring, or happy film. For ethical reasons, participants were informed the upsetting film would involve a car accident.

#### Phase 1

**Fig. 1:**
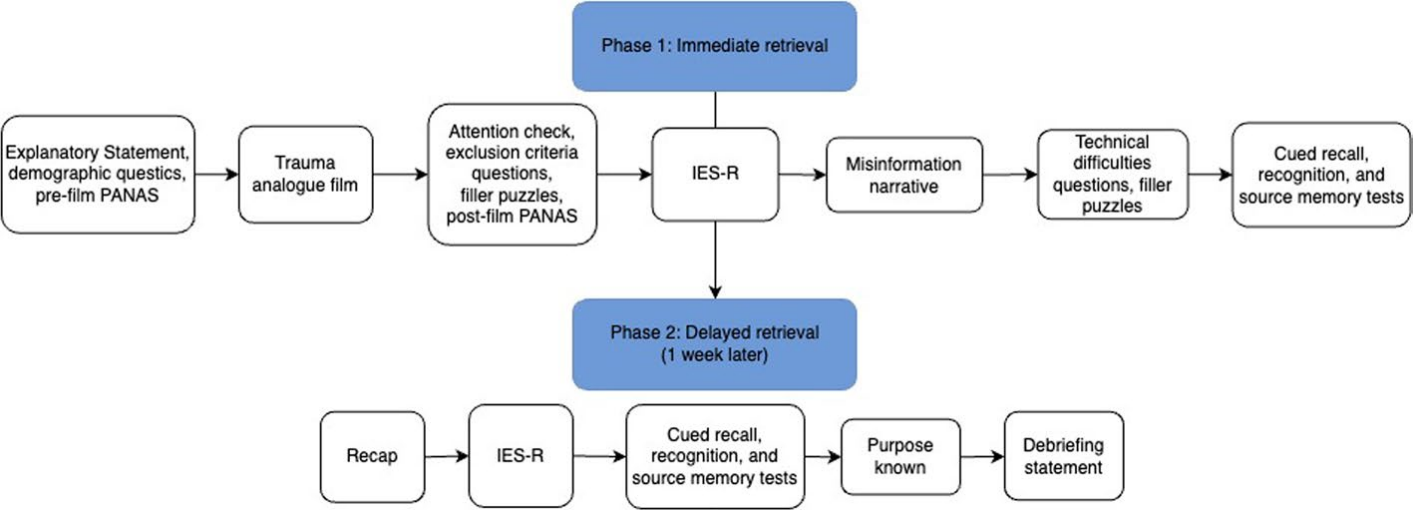
Study procedure

Participants read the explanatory statement, answered demographic questions, and completed the pre-film PANAS. All participants then watched the film clip. They were asked whether they had seen the film before, experienced any technical difficulties, or had paused or skipped the video at any point. They then answered two attention check questions. Participants were excluded on these questions as outlined above. After filler puzzles lasting two minutes, participants completed the post-film PANAS and the IES-R. Participants were then randomly allocated to listen to one of three audio misinformation narratives. They completed another round of filler logical questions lasting two minutes. Then they completed the cued recall test with confidence ratings, recognition test, and source memory test. At the end of Phase 1, participants were asked whether they completed the phase in one sitting and were told they would be contacted in one week for Phase 2. The study’s procedure is displayed in Fig. 1.

#### Phase 2

One week later, participants were recapped of the study’s content (i.e. that they previously watched a film about a car crash and answered some questions) to reorient them to the study. They once again completed the IES-R for any film-related distress reactions experienced over the past week. Participants then completed the memory tests in the same order as in Phase 1. Participants were then asked what they thought the purpose of the study was and whether they completed Phase 2 in a single sitting. Finally, all participants read a debriefing statement and received compensation for their time.

### Data coding and analysis

All memory test responses were binary coded for accuracy (0 = incorrect, 1 = correct). To code qualitative cued recall responses, a coding scheme was created before data collection. Inter-rater reliability for coding of cued recall questions was checked for 25% of the sample (*n* = 60). LJ and PS completed coding and were both blind to item type. Cohen’s kappa showed excellent agreement (*K* = 0.94). The coding scheme for source memory and cued recall responses can be accessed via OSF.

Generalised linear mixed models (*GLMMs*) were performed using the *lme4* package (Bates et al., 2015) in RStudio, using maximum likelihood estimation. *GLMM*s are a suitable analysis when the outcome variable does not follow a normal distribution (Miller et al., 2020; Tuerlinckx et al., 2006) and for repeated measures data (Bono et al., 2021). The models also handle missing data well, as only data for missing time points are excluded, rather than entire data for non-returning participants (de Melo et al., 2022). We ran separate models for the hypotheses, using the same process of analysis. The outcome variable for all models was binary accuracy, thus applying the logit/logistic link function to analyses. Given our focus on performance on misled items, we set misled items as our reference level for all analyses. For Hypothesis 1, we examined whether item type was a significant predictor of accuracy, specifically whether accuracy was lower for misled items than for non-misled (control and consistent) items. Item type was fitted as a fixed effect. For Hypotheses 2 and 3, we explored whether distress interacted with item type and retrieval time to affect memory accuracy through a three-way interaction between distress, item type, and time. For every model, participants were set as the random effect to control for unmeasured participant differences that may influence accuracy. The same process of analysis was applied to our preregistered exploratory analyses on the effects of types of distress (avoidance, intrusions, and hyperarousal) on memory accuracy. Given the scope of this paper and detail required, other preregistered exploratory variables (confidence, central/peripheral items, and film relevance) have not been included.

In addition to computing *GLMMs*, *p*-values were computed using the *afex* package (Singmann & Kellen, 2019), odds ratios using *broom.mixed* (Bolker et al., 2022), and graphs and tables of models using *sjPlot* (Lüdecke et al., 2023) and *ggplot2* (Wickham et al., 2016). Mean IES-R total and subscale scores were centred around their average to interpret interactions. Cued recall, recognition, and source memory results are discussed separately. Odds ratios (OR) are used as a measure of effect size and indicate the odds of being accurate on control or consistent items compared to misled items. For Hypothesis 2 and exploratory analyses, the OR shows how the relationship between distress and accuracy differs, for each item type. For example, if comparing consistent and misled items, an OR greater than 1 suggests that for every unit increase in distress, the odds of a correct response on consistent items increased compared to the odds for misled items (i.e. a larger misinformation effect). An OR less than 1 suggests that for each unit increase in distress, the odds of a correct response decrease for consistent items compared to misled items (i.e. a smaller misinformation effect). The plotted graphs show the probability of accuracy for each item type.

## Results

As participants who did not return for Phase 2 were retained in the analyses, the descriptive statistics of the full sample are provided in Table 1. Descriptive statistics for participants who completed both phases are presented in Supplementary Materials. Out of the 199 participants who completed Phase 2, few participants indicated knowing the purpose of the study (*n* = 12, 6%). Knowing the purpose did not significantly affect accuracy. As shown in Table 1, the film led to a significant increase in the level of negative affect (PANAS) reported by participants (*p* < 0.001) and decrease in self-reported positive affect (*p* < 0.001).

**Table 1:** Descriptive statistics for participants who completed Phase 1 (*n* = 243)

Variable	Mean (*SD*) (unless specified)	Range
Age (years)	40.15 (10.96)	20–72
Gender^a^ (*n*)	122:117:2:2	
Education^b^ (*n*)	37:41:108:50:3:4	
Culture^c^ *n*	3:4:29:14:177:1	
PANAS Positive affect		
Before film	29.83 (9.72)	10–50
After film	27.16 (9.61)	10–50
PANAS Negative affect		
Before film	13.42 (6.61)	10–45
After film	17.77 (7.95)	10–49
IES-R Total (Phase 1)	3.57 (2.62)	0–10.32
Avoidance	1.20 (0.87)	0–3.43
Intrusions	1.31 (1.07)	0–4
Hyperarousal	1.07 (0.95)	0–3.80
IES-R Total (Phase 2)	1.81 (2.21)	0–10.56
Avoidance	0.84 (0.86)	0–3.71
Intrusions	0.57 (0.83)	0–4
Hyperarousal	0.40 (0.75)	0–3.80

^a^Woman; man; non-binary/non-conforming; prefer not to say

^b^Secondary; post-secondary; undergraduate degree; postgraduate degree; other; prefer not to say

^c^American Indian or Alaska Native; Asian; Black or African-American; Hispanic or Latino; White or Caucasian; not specified

### Hypothesis 1: Misinformation effect

**Fig. 2:**
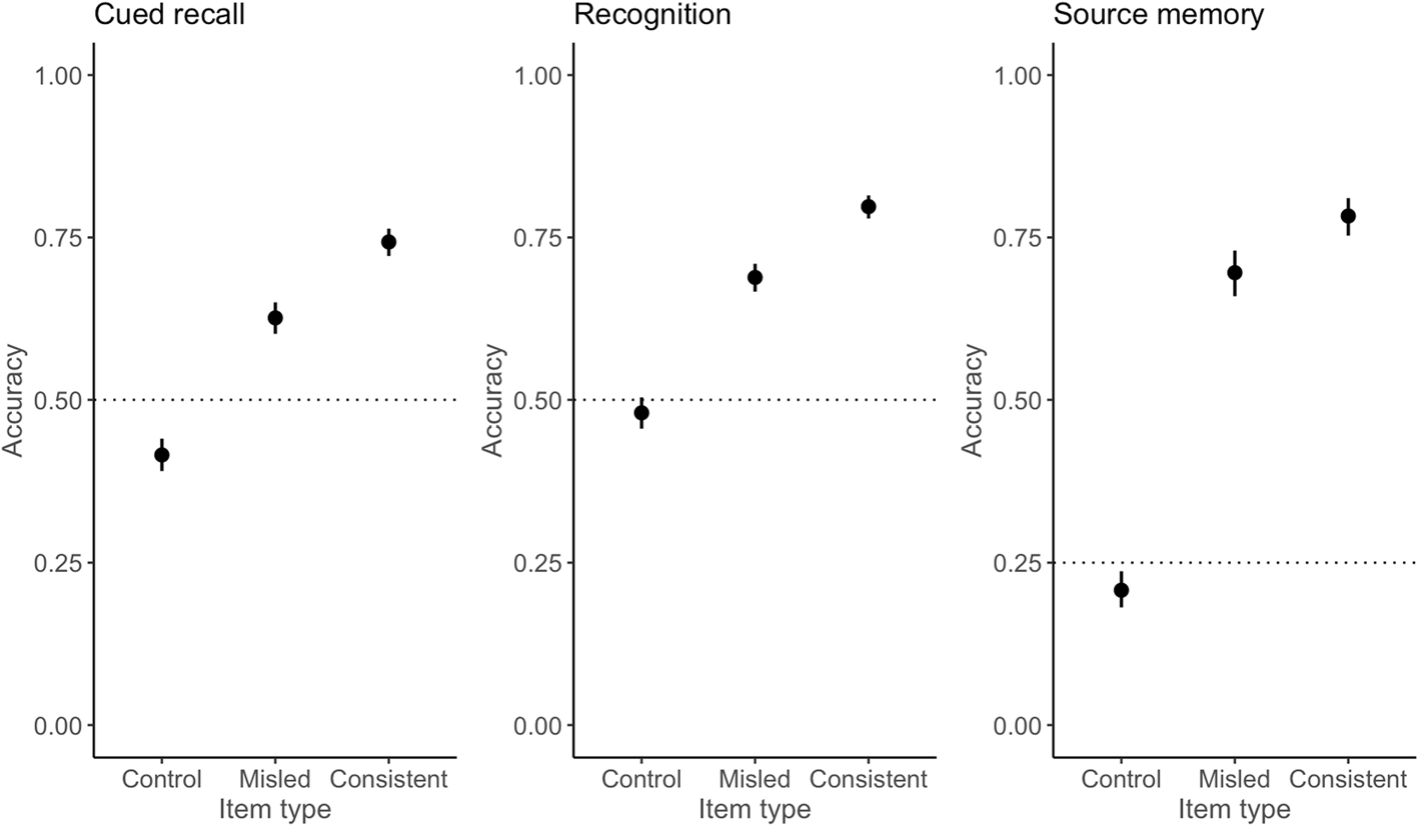
Predicted probabilities of accuracy for misled items for cued recall, recognition, and source memory. *Note.* Dashed line indicates chance performance on each test

As predicted, across the three memory tests, participants showed significantly lower accuracy on misled items than control items (all *p* < 0.001) and consistent items (all *p* < 0.001). The predicted probabilities of accuracy for each item type and test type are shown in Fig. 2. Odds ratios for the interactions and respective *p*-values are provided in Table 2. The odds of being correct on control items were about twice as high as on misled items on cued recall (OR 2.35) and recognition (OR 2.39). For consistent items, the odds of being correct were four times higher than on misled items on the cued recall test (OR 4.06) and recognition test (OR 4.27).

the distress × item type interactions are discusse

**Table 2:** Effect of misinformation and total distress (IES-R) on accuracy on cued recall, recognition, and source memory tests: parameter estimates of the GLMMs

Predictors	Cued recall			Recognition			Source memory		
	Estimates [95% CI]	O.R. [95% CI]	*p*	Estimates [95% CI]	O.R. [95% CI]	*p*	Estimates [95% CI]	O.R. [95% CI]	*p*
*Misinformation effect*									
Intercept	− 0.34 [− 0.44 to − 0.24]	0.71 [0.64–0.79]	** < 0.001**	− 0.08 [0.18–0.02]	0.92 [0.84–1.02]	0.100	− 1.34 [− 1.51 to − 1.17]	0.26 [0.22–0.31]	** < 0.001**
Control	0.86 [0.74–0.97]	2.35 [2.10–2.63]	** < 0.001**	0.87 [0.76–0.99]	2.39 [2.14–2.68]	** < 0.001**	2.17 [2.02–2.32]	8.73 [7.51–10.14]	** < 0.001**
Consistent	1.40 [1.28–1.52]	4.06 [3.60–4.58]	** < 0.001**	1.45 [1.33–1.57]	4.27 [3.77–4.83]	** < 0.001**	2.62 [2.47–2.78]	13.77 [11.79– 16.09]	** < 0.001**
*Distress and misinformation effect*									
Intercept	− 0.38 [− 0.51 to − 0.25]	0.69 [0.60–0.78]	** < 0.001**	− 0.10 [− 0.22 to 0.02]	0.90 [0.80–1.02]	0.106	− 1.09 [− 1.29 to − 0.90]	0.33 [0.28–0.41]	** < .001**
Control	0.95 [0.79–1.10]	2.58 [2.20–3.02]	** < 0.001**	1.04 [0.88–1.20]	2.83 [2.41–3.32]	** < 0.001**	2.11 [1.91–2.32]	8.28 [6.75–10.17]	** < 0.001**
Consistent	1.55 [1.38–1.72]	4.72 [3.99–5.58]	** < 0.001**	1.58 [1.41–1.76]	4.87 [4.09–5.79]	** < 0.001**	2.55 [2.33–2.76]	12.76 [10.31–15.80]	** < 0.001**
Delayed retrieval	0.09 [− 0.09 to 0.26]	1.09 [0.92–1.30]	0.330	0.07 [− 0.11 to 0.24]	1.07 [0.90–1.27]	0.453	− 0.57 [− 0.81 to − 0.34]	0.56 [0.44–0.72]	** < 0.001**
IES-R	− 0.03 [− 0.07–0.01]	0.97 [0.93–1.01]	0.196	− 0.01 [− 0.05 to 0.03]	0.99 [0.96–1.03]	0.781	0.05 [− 0.01 to 0.11]	1.05 [0.99–1.12]	0.080
Control × IES-R	0.00 [− 0.04 to 0.05]	1.00 [0.96–1.05]	0.887	− 0.01 [− 0.06 to 0.04]	0.99 [0.95–1.04]	0.693	− 0.11 [− 0.17 to − 0.05]	0.89 [0.84–0.95]	** < 0.001**
Consistent × IES-R	− 0.05 [− 0.10 to − 0.00]	0.95 [0.90–1.00]	**0.031**	− 0.05 [− 0.10 to − 0.00]	0.95 [0.91–1.00]	**0.033**	− 0.15 [− 0.21 to − 0.09]	0.86 [0.81–0.91]	** < 0.001**

Recall that the source memory test comprised 36 statements, including 18 correct statements (i.e. correct detail from the video) and 18 incorrect statements (i.e. the misleading detail). Table 3 shows the proportion of each source memory response (i.e. video only, summary only, video and summary, I don’t know, no memory) by participants on correct and incorrect statements by each item type. The correct responses are highlighted in bold. Interestingly, for incorrect statements, none of the participants attributed the misleading details to the video only (correctly so). Instead, participants were most likely to attribute the misleading detail to ‘both’ sources, or claim they had ‘no memory’ of it. It is also worth noting that even for correct statements, participants rarely attributed a detail to ‘video only’ even when this was the correct response, and instead tended to attribute it to ‘both’ sources. This was not the case for consistent items, where most participants picked the correct answer. Overall, although participants showed very low accuracy on misled items, this may also reflect a general tendency to pick the incorrect but safer option (i.e. ‘both’) within the sample.

**Table 3:** Rate of responding ‘video only,’ ‘summary only,’ ‘both,’ ‘I don’t know,’ and ‘no memory’ by each item type and statement type

Statement type	Item type	Response				
		‘Video only’	‘Summary only’	‘Both’	‘I don’t know’	‘No memory’
Correct statements	Control	**0.02**	0.05	0.55	0.17	0.21
	Misled	**0.01**	0.05	0.43	0.14	0.36
	Consistent	0.01	0.13	**0.62**	0.10	0.14
Incorrect statements	Control	0	0.07	0.19	0.21	**0.53**
	Misled	0	**0.22**	0.33	0.13	0.31
	Consistent	0	0.05	0.16	0.16	**0.63**

Correct responses are in bold

Note that in all subsequent analyses of the source monitoring test data, ‘I don’t know’ responses were coded as ‘not applicable.’ Given our focus on how well people can recall the source of a suggested detail, only accuracy on incorrect statements was further analysed. Thus, for all analyses regarding the source memory test, the outcome variable was accuracy on incorrect statements.

### Hypotheses 2 and 3: The association between distress (total IES-R) and accuracy for misled items over time

**Fig. 3:**
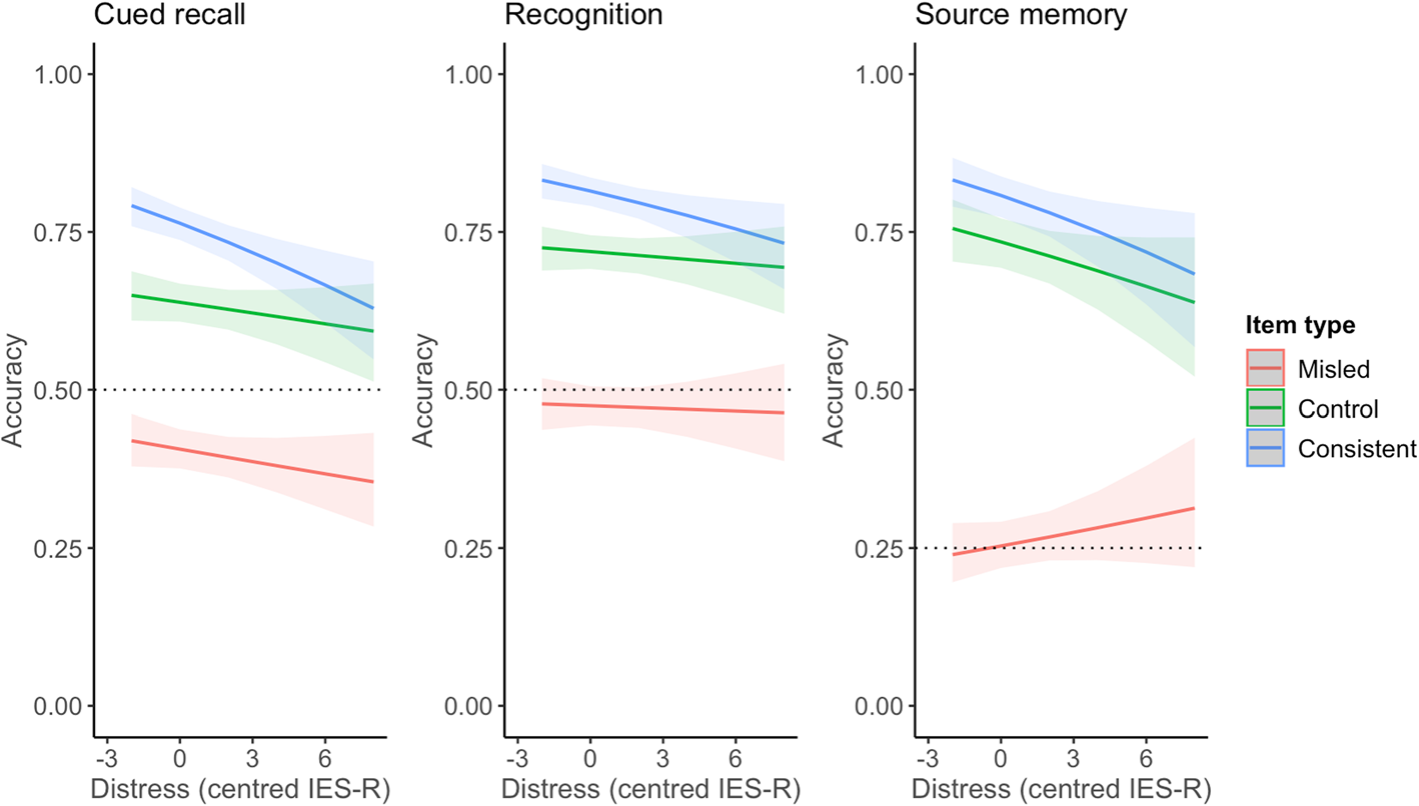
Depiction of interaction between IES-R symptoms × item type for cued recall, recognition, and source memory tests. *Note.* Dashed line indicates chance performance on each test

Figure 3 shows the predicted probabilities of accuracy for each item type as distress increased in the sample. As distress scores were centred, 0 represents the average IES-R scores reported in the sample across both phases. The misinformation effect found in Hypothesis 1 is clearly seen in the difference in accuracy between the item types, with accuracy on misled items being lowest. Across all tests, this difference is smaller for participants who reported higher levels of distress compared to those who reported lower distress. As can be seen from the steep drop in accuracy for consistent items, higher levels of distress were associated with especially impaired memory for consistent items, while accuracy for misled items changed at a slower rate similar to control items. For source memory, greater distress levels were associated with improved accuracy for misled items. In sum, distress was associated with a smaller misinformation effect. The coefficients for the interactions are discussed below.

Contrary to our expectations, none of our models showed a significant three-way interaction between total distress, item type, and retrieval time, all *p*s > 0.05. Thus, the impact of distress on accuracy did not depend on retrieval time. Given our interest in the relationship between distress and misinformation, we conducted models inspecting two-way interactions between each variable (i.e. item type × distress, item type × retrieval time, distress × retrieval time). Only relevant results on the distress × item type interactions are discussed below and reported in Table 2. Results for the interactions including retrieval time (i.e. item type × retrieval time and distress × retrieval time) are provided in Supplementary Materials.

#### Cued recall

A significant interaction between item type and distress was found (*p* = 0.04) when comparing misled and consistent items. Each unit increase in distress was associated with lower odds of being accurate on consistent items (OR 0.95, 95% CI [0.90, 1.00]), compared to on misled items. In other words, higher levels of distress were associated with a reduction in the misinformation effect. A difference was not found when comparing misled and control items (OR 1.00, 95% CI [0.93, 1.05]). As a main effect, distress did not significantly affect accuracy (*p* = 0.20).

#### Recognition

Similar to cued recall performance, each unit increase in distress was associated with lower odds of being accurate on consistent items compared to misled items (OR 0.95, 95% CI [0.91, 1.00]). A difference was not found comparing misled and control items (OR 0.99, 95% CI [0.95, 1.04]). However, this two-way interaction between item type and distress was not significant (*p* = 0.09). As a main effect, distress was not significantly associated with accuracy (*p* = 0.78).

#### Source memory

Recall that for source memory, the outcome variable was accuracy on incorrect statements. A significant interaction between item type and total distress was found (*p* < 0.001). Each unit increase in distress was associated with lower odds of being accurate on control (OR 0.89, 95% CI [0.84, 0.95]) and consistent (OR 0.86, 95% CI [0.81, 0.91]) items compared to misled items. As a main effect, distress was not significant (*p* = 0.08).

### Exploratory analyses

We were interested in how the three different symptoms of distress (avoidance, intrusions, and hyperarousal) can impact the misinformation effect over time and how this may change across test types. To understand whether the effect of these symptoms on the misinformation effect changed over time, we conducted exploratory analyses to inspect for a three-way interaction between symptom × item type × retrieval time. When non-significant, we conducted a model inspecting a two-way interaction between symptom × item type (same as above).

#### Cued recall

**Fig. 4:**
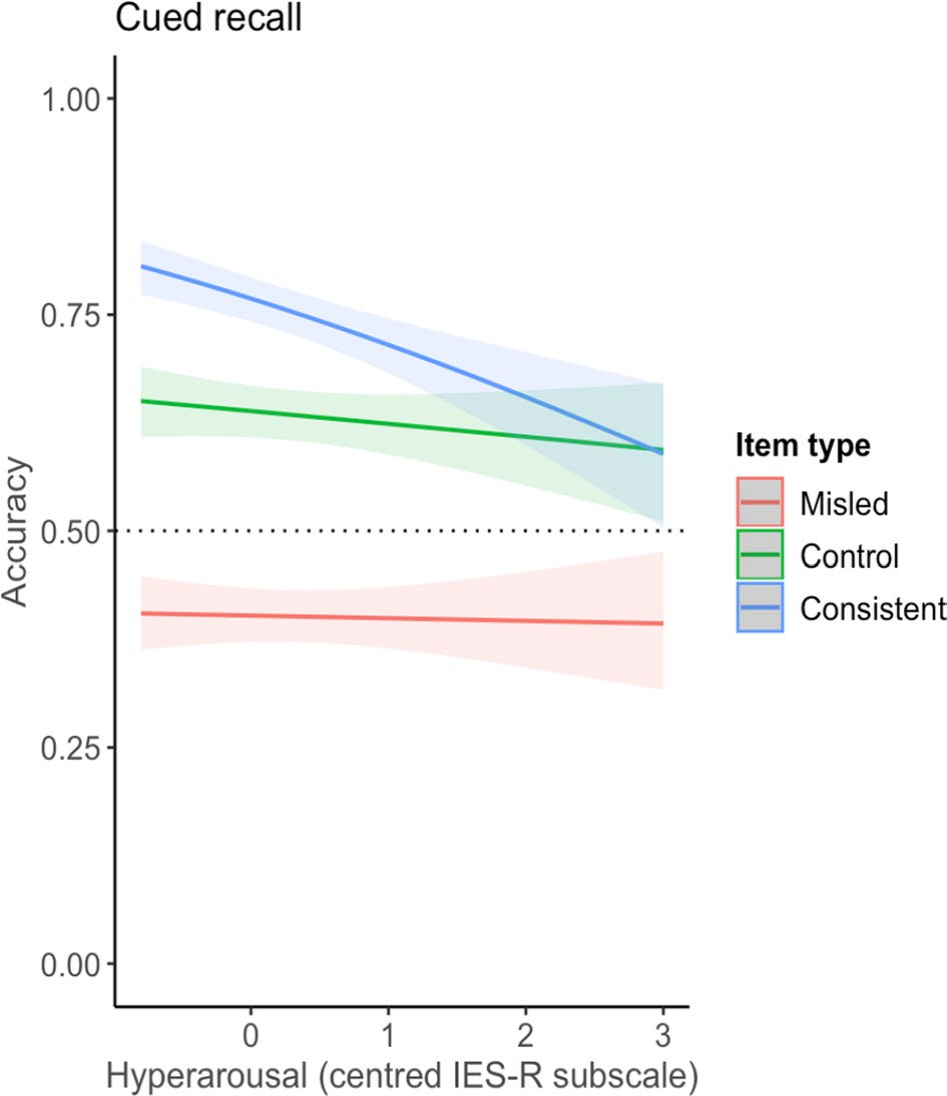
Depiction of interaction between hyperarousal symptoms × item type for cued recall memory. *Note.* Dashed line indicates chance performance

No significant two-way interaction was found between avoidance symptoms and item type (*p* = 0.31). The two-way interaction between intrusion symptoms and item type was not significant (*p* = 0.09), with non-significant odds ratios comparing misled to control (OR 1.09, 95% CI [0.97, 1.23]) or consistent (OR 0.96, 95% CI [0.85, 1.08]) items. Figure 4 shows that greater hyperarousal symptoms were associated with a steep reduction in accuracy for consistent items. Participants reporting higher levels of hyperarousal symptoms showed a smaller misinformation effect (hyperarousal symptom × item type interaction, *p* < 0.001). Each unit increase in hyperarousal symptoms was associated with lower odds of reporting a correct response on consistent items compared to on misled items (OR 0.77, 95% CI [0.67,0.88]) (Fig. 4). A difference between control and misled items was not found (OR 0.95, 95% CI [0.83,1.09]). Table 4 presents the coefficients for the cued recall exploratory analyses, showing estimates for the selected two-way interaction models for avoidance, intrusions, and hyperarousal.

**Table 4:** Effect of types of distress (avoidance, intrusions, and hyperarousal), item type, and retrieval time on cued recall memory accuracy: parameter estimates of selected GLMMs

Predictors	Avoidance			Intrusions			Hyperarousal		
	Estimates [95% CI]	O.R. [95% CI]	*p*	Estimates [95% CI]	O.R. [95% CI]	*p*	Estimates [95% CI]	O.R. [95% CI]	*p*
Intercept	− 0.39 [− 0.52 to − 0.26]	0.68 [0.60–0.77]	** < 0.001**	− 0.37 [− 0.49 to − 0.24]	0.69 [0.61–0.79]	** < 0.001**	− 0.39 [− 0.52 to − 0.27]	0.67 [0.59–0.77]	** < 0.001**
Control	0.96 [0.80–1.11]	2.60 [2.23–3.04]	** < 0.001**	0.92 [0.76–1.08]	2.51 [2.15–2.94]	** < 0.001**	0.96 [0.80–1.12]	2.62 [2.24–3.07]	** < 0.001**
Consistent	1.52 [1.36–1.69]	4.59 [3.89–5.40]	** < 0.001**	1.52 [1.35–1.69]	4.59 [3.88–5.43]	** < 0.001**	1.59 [1.43–1.76]	4.93 [4.16–5.84]	** < 0.001**
Delayed retrieval	0.12 [− 0.05 to 0.28]	1.13 [0.96–1.33]	0.157	0.05 [− 0.13 to 0.23]	1.05 [0.88–1.26]	0.582	0.12 [− 0.05 to 0.30]	1.13 [0.95–1.35]	0.167
Distress type (main effect)	− 0.06 [− 0.18 to 0.06]	0.94 [0.83–1.06]	0.339	− 0.11 [− 0.21 to − 0.01]	0.90 [0.81–1.00]	**0.040**	− 0.01 [− 0.13 to 0.10]	0.99 [0.88–1.11]	0.828
Control × Distress type	− 0.03 [− 0.16 to 0.10]	0.97 [0.85–1.11]	0.642	0.09 [− 0.03 to 0.21]	1.09 [0.97–1.23]	0.149	− 0.05 [− 0.18 to 0.08]	0.95 [0.83–1.09]	0.454
Consistent × Distress type	− 0.11 [− 0.24 to 0.03]	0.90 [0.78–1.03]	0.130	− 0.04 [− 0.17 to 0.08]	0.96 [0.85–1.08]	0.480	− 0.27 [− 0.40 to 0.13]	0.77 [0.67–0.88]	** < .001**

#### Recognition

**Fig. 5:**
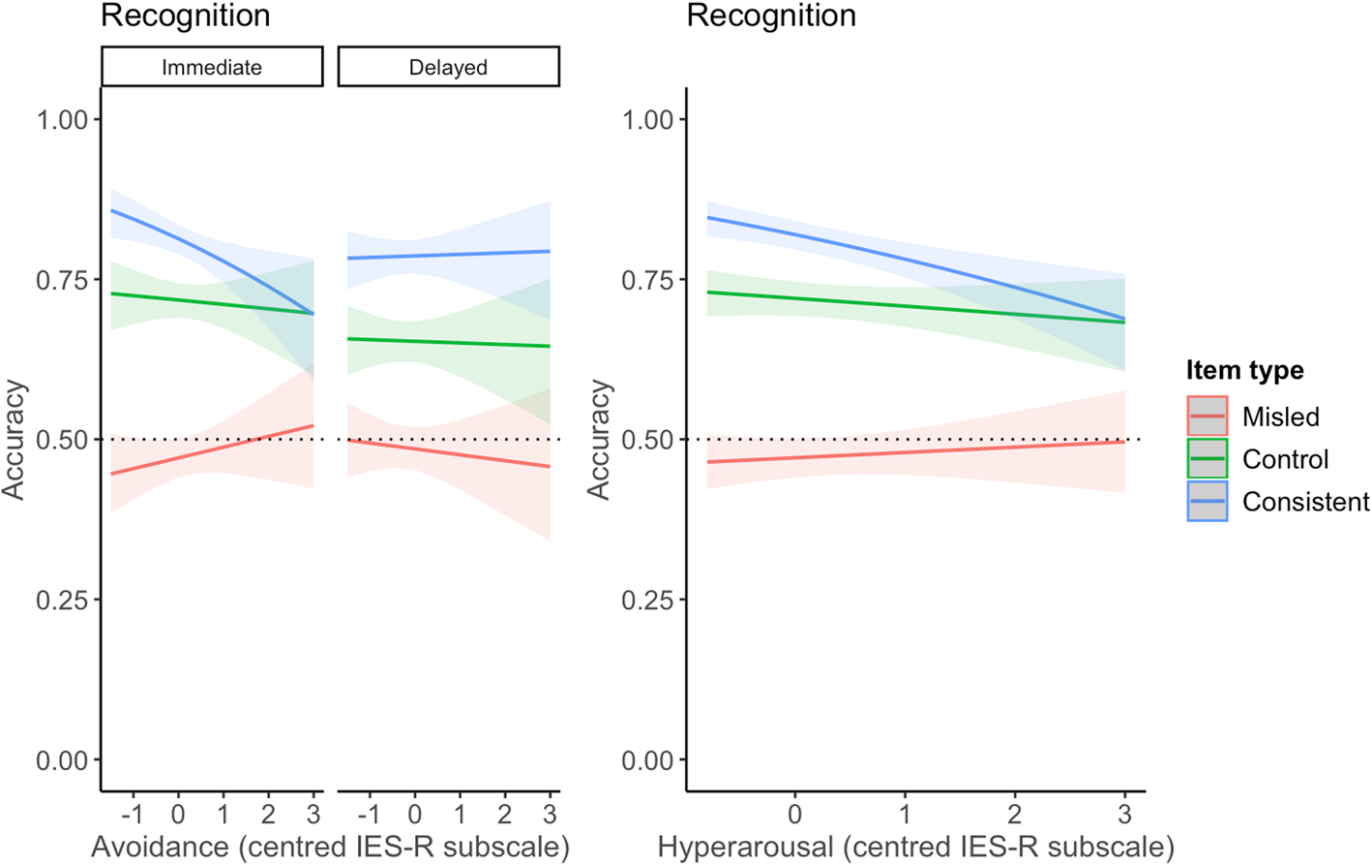
Depiction of interaction between Avoidance × Item Type × Retrieval Time, and Hyperarousal × Item Type for Recognition Memory Accuracy. *Note.* Dashed line indicates chance performance

For avoidance symptoms, there was some evidence that the relationship between avoidance and item type accuracy changed depending on retrieval time. When comparing misled and consistent items, greater avoidance was associated with a smaller misinformation effect in immediate retrieval and a larger misinformation effect in delayed retrieval (OR 1.40, 95% CI [1.05, 1.86] (Fig. 5). In immediate retrieval, participants reporting greater avoidance of the film showed better memory for misled items and worse memory for consistent items. In delayed retrieval, participants reporting greater avoidance showed worse memory for misled items and slightly better memory for consistent items. A difference was not found when comparing misled and control items (OR 1.13, 95% CI [0.87, 1.48]). However, this three-way interaction between avoidance symptoms, item type, and retrieval time was not significant (*p* = 0.07). For intrusion symptoms, a two-way interaction between intrusions and item type was not significant (*p* = 0.54). For hyperarousal symptoms, a two-way interaction was significant (*p* < 0.001) comparing misled and consistent items. Higher hyperarousal symptoms were associated with a smaller misinformation effect, where each unit increase in hyperarousal symptoms was associated with lower odds of being accurate on consistent items compared to misled items (OR 0.76, 95% CI [0.66, 0.87]) (Fig. 5). A difference between misled and control items was not found (OR 0.91, 95% CI [0.80–1.04]). Table 5 presents the coefficients for the recognition exploratory analyses, showing estimates for the three-way interaction model for avoidance, and two-way interaction model for intrusions and hyperarousal.

**Table 5:** Effect of types of distress (avoidance, intrusions, and hyperarousal), item type, and retrieval time on recognition memory accuracy: parameter estimates of selected GLMMs

Predictors	Avoidance			Intrusions			Hyperarousal		
	Estimates [95% CI]	O.R. [95% CI]	*p*	Estimates [95% CI]	O.R. [95% CI]	*p*	Estimates [95% CI]	O.R. [95% CI]	*p*
Intercept	− 0.12 [− 0.24 to 0.00]	0.89 [0.79 to 1.00]	0.058	− 0.08 [− 0.21 to 0.04]	0.92 [0.81–1.04]	0.182	− 0.12 [− 0.24 to 0.01]	0.89 [0.79–1.01]	0.066
Control	1.05 [0.89–1.21]	2.85 [2.43–3.35]	** < 0.001**	1.02 [0.85–1.18]	2.76 [2.35–3.24]	** < 0.001**	1.06 [0.90–1.22]	2.89 [2.46–3.40]	** < 0.001**
Consistent	1.59 [1.41–1.76]	4.89 [4.12–5.82]	** < 0.001**	1.54 [1.37–1.72]	4.67 [3.93–5.56]	** < 0.001**	1.63 [1.45–1.81]	5.10 [4.28–6.09]	** < 0.001**
Delayed retrieval	0.06 [− 0.11 to 0.22]	1.06 [0.90–1.24]	0.496	0.03 [− 0.14 to 0.21]	1.03 [0.87–1.23]	0.701	0.10 [− 0.07 to 0.28]	1.11 [0.93–1.32]	0.244
Distress type (main effect)	0.07 [− 0.07 to 0.20]	1.07 [0.94–1.22]	0.323	− 0.07 [− 0.16 to 0.03]	0.94 [0.85–1.03]	0.192	0.03 [− 0.08 to 0.14]	1.03 [0.93–1.16]	0.558
Control × Distress type	− 0.10 [− 0.28 to 0.08]	0.90 [0.76–1.08]	0.272	0.05 [− 0.07 to 0.17]	1.05 [0.93–1.18]	0.409	− 0.09 [− 0.23 to 0.04]	0.91 [0.80–1.04]	0.169
Consistent × Distress type	− 0.28 [− 0.48 to − 0.09]	0.75 [0.62–0.91]	**0.004**	− 0.02 [− 0.15 to 0.11]	0.98 [0.86–1.11]	0.752	− 0.27 [− 0.41 to − 0.14]	0.76 [0.66–0.87]	** < 0.001**
Control × Distress type × Delayed retrieval	− 0.13 [− 0.14 to 0.39]	1.13 [0.87–1.48]	0.350						
Consistent × Distress type × Delayed retrieval	0.34 [0.05–0.62]	1.40 [1.05–1.86]	**0.021**						

#### Source memory

**Fig. 6:**
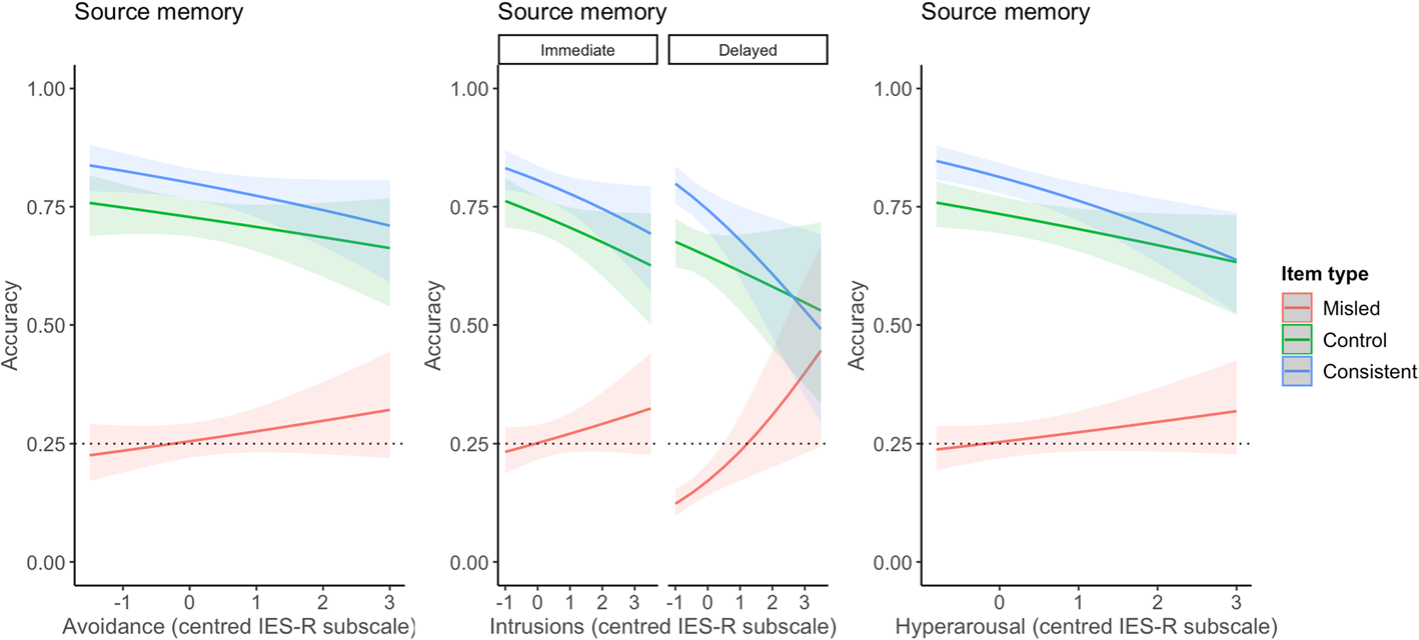
Depiction of Interaction between Avoidance × Item Type, Intrusions × Item Type × Retrieval Time, and Hyperarousal × Item Type for Source Memory Accuracy. *Note*. Dashed line indicates chance performance

Recall that the outcome variable for all source memory analyses was accuracy on incorrect statements. A two-way interaction between avoidance symptoms and item type was found (*p* = 0.01), where higher avoidance symptoms was associated with a smaller misinformation effect (Fig. 6). Each unit increase in avoidance was paired with lower odds of being correct on control (OR 0.81, 95% CI [0.68, 0.96]) and consistent items (OR 0.76, 95% CI [0.64, 0.91]), compared to misled items. A three-way interaction between intrusion symptoms, retrieval time, and item type was found (*p* = 0.04) when comparing misled and consistent items. As also shown in Fig. 6, each unit increase in intrusion symptoms was associated with greater accuracy on misled items and lower accuracy on consistent items. This difference became greater during delayed retrieval, as higher intrusion symptoms were associated with improving accuracy on misled items and even poorer accuracy on consistent items (OR 0.65, 95% CI [0.47, 0.90]). This difference was not found when comparing misled and control items (OR 0.76, 95% CI [0.55, 1.04]). Thus, over time, greater intrusions of the film were associated with an improved ability to discriminate against misled items but poorer accuracy for consistent items. A two-way interaction between hyperarousal symptoms and item type was significant (*p* < 0.001), showing that higher hyperarousal symptoms were associated with lower odds of accuracy on control (OR 0.77, 95% CI [0.65, 0.91]) and consistent items (OR 0.66, 95% CI [0.56, 0.79]), compared to misled items (Fig. 6). In summary, higher levels of all three symptoms of distress were associated with improved source memory for misled items, and intrusive symptoms were associated with a greater improvement over time. Table 6 provides the coefficients for the source memory exploratory analyses, showing estimates for the two-way interaction model for avoidance, three-way interaction model for intrusions, and two-way interaction model for hyperarousal.

**Table 6:** Effect of types of distress (avoidance, intrusions, and hyperarousal), item type, and retrieval time on source memory accuracy: parameter estimates of selected GLMMs

Predictors	Avoidance			Intrusions			Hyperarousal		
	Estimates [95% CI]	O.R. [95% CI]	*p*	Estimates [95% CI]	O.R. [95% CI]	*p*	Estimates [95% CI]	O.R. [95% CI]	*p*
Intercept	− 1.07 [− 1.26 to − 0.88]	0.34 [0.28–0.41]	** < 0.001**	− 1.09 [− 1.29 to − 0.90]	0.34 [0.28–0.41]	** < 0.001**	− 1.08 [− 1.27 to − 0.89]	0.34 [0.28–0.41]	** < 0.001**
Control	2.06 [1.86–2.26]	7.83 [6.41–9.56]	** < 0.001**	2.11 [1.90–2.32]	8.26 [6.69–10.18]	** < 0.001**	2.10 [1.89–2.30]	8.15 [6.64– 10.02]	** < 0.001**
Consistent	2.46 [2.26–2.67]	11.73 [9.55–14.42]	** < 0.001**	2.52 [2.30–2.73]	12.37 [9.96–15.37]	** < 0.001**	2.55 [2.33–2.76]	12.78 [10.31–15.84]	** < 0.001**
Retrieval time	− 0.65 [− 0.87 to − 0.43]	0.52 [0.42–0.65]	** < 0.001**	− 0.49 [− 0.73 to − 0.25]	0.61 [0.48–0.78]	** < 0.001**	− 0.60 [− 0.84 to − 0.35]	0.55 [0.43–0.70]	** < 0.001**
Distress type main effect	0.11 [− 0.06 to 0.28]	1.11 [0.94–1.32]	0.220	0.10 [− 0.05 to 0.25]	1.11 [0.96–1.28]	0.174	− 0.11 [− 0.05 to 0.27]	1.11 [0.95–1.30]	0.186
Control × Distress type	− 0.21 [− 0.38 to 0.04]	0.81 [0.68–0.96]	**0.016**	− 0.25 [− 0.43 to − 0.07]	0.78 [0.65–0.93]	**0.007**	− 0.26 [− 0.43 to 0.10]	0.77 [0.65–0.91]	**0.002**
Consistent × Distress type	− 0.27 [− 0.45 to − 0.10]	0.76 [0.64–0.91]	**0.002**	− 0.28 [− 0.46 to − 0.09]	0.76 [0.63–0.91]	**0.003**	− 0.41 [− 0.58 to − 0.23]	0.66 [0.56–0.79]	** < 0.001**
Control × Distress type × Delayed retrieval				− 0.28 [− 0.60 to − 0.04]	0.76 [0.55–1.04]	0.085			
Consistent × Distress type × Delayed retrieval				− 0.43 [− 0.76 to − 0.10]	0.65 [0.47–0.90]	**0.011**			

## Discussion

This study investigated how distress was associated with the misinformation effect for an analogue trauma event. In support of Hypothesis 1, a clear misinformation effect was found, showing that analogue trauma material is susceptible to distortion. Hypothesis 2 was partly supported; participants who reported higher distress in response to the film showed a smaller misinformation effect across all tests, owing to especially poor memory for consistent items. Hypothesis 3 was not supported, as we did not find the effect of total distress on the misinformation effect to vary as a function of time. We also found that certain distress symptoms may be differently implicated in memory tests, with avoidance being associated with recognition and source memory, intrusions being associated with source memory, and hyperarousal being associated with each memory task. The association between avoidance and recognition was in the direction we expected. Across all tasks, distressed participants showed the smallest misinformation effect when source memory was assessed.

More distressed participants showed especially poor memory for consistent items compared to misled items, suggesting symptoms of distress may not necessarily be associated with the distorting influence of misinformation but may impede memory otherwise. Reduced performance on consistent items fits with the literature on stress and memory that shows stress to lead to a habitual memory system where memory of the event is not ‘updated’ with new information (Quaedflieg & Schwabe, 2018). Thus, distressed participants’ memory may have been guarded from the facilitating effect of repeated, true information, thus being associated with a faster reduction in accuracy compared to less distressed participants. This explanation would also see improved accuracy for misled items for higher levels of distress, as memory would also be guarded from the distorting effect of incoming misinformation. This result was found when symptoms of distress were measured (see Figs. 5, 6) but not total distress (see Fig. 3) or cued memory (see Fig. 4). However, accuracy on control items did not improve with higher distress, as would be expected from this account. Another account of these findings is discussed by Moore and Zoellner (2012) who found similar results, where suppression (an avoidance strategy) was associated with poorer memory accuracy and reduced misinformation. Drawing on the cognitive load model (Lavie et al., 2004), the authors posited that suppression, being a cognitively effortful task, may have directed attention towards information outside of the task at hand, such as source information. This would see a reduction in misinformation, as source information can reduce distortion (Johnson et al., 1993), while also reducing accuracy as attentional focus is broadened (Moore & Zoellner, 2012). This can also explain why participants reporting high distress showed the smallest misinformation effect on source memory responses within our study.

There was some evidence of a time-dependent effect of avoidance on the misinformation effect in recognition memory, adding to some previous findings on this relationship. We found that avoidance of reminders of the film was associated with a smaller misinformation effect on recognition memory measured immediately after the film. This is consistent with Moore and Zoellner (2012) and Monds et al. (2013). Importantly, the effect of avoidance changed in the opposite direction over time; higher symptoms were associated with poorer recognition accuracy on misled items at delayed retrieval. This replicates findings by Monds et al. (2013), who found post-film avoidance was associated with less endorsement of misinformation while persistent (one week) avoidance showed the opposite effect. However, Moore and Zoellner (2012) found avoidance was associated with a smaller misinformation effect when retrieval was delayed by 48 h. It is likely that the debilitating effects of avoidance on the misinformation effect commence post-48 h of the event, suggesting a time-dependent effect of avoidance on the misinformation effect that warrants further investigation. Attempts at avoidance may be temporarily successful but counterproductively increase the accessibility of the to-be-avoided thought in the long term, a phenomenon termed the ‘rebound’ effect (Nixon et al., 2009; Wegner et al., 1991; Wenzlaff & Wegner, 2000). This would see a smaller misinformation effect in the short term, as misinformation is suppressed along with accurate details, and a larger misinformation effect in the long term, as these details ‘rebound’ not only reminding of the true information but also of misinformation. This pattern of findings may also reflect the continued use of the habitual memory system associated with stress (Quaedflieg & Schwabe, 2018) as over time, the memory of the stressful event is left unintegrated, and gaps may be filled with misinformation. Note that these findings were not repeated by Monds et al. (2016), where immediate memory testing occurred before self-reported measures of distress, questioning whether timing of the attention to distress states plays a role in how distress affects memory and misinformation. Importantly, the three-way interaction between avoidance, item type, and retrieval time was not significant (*p* = 0.07). Given that this was an exploratory analysis and a time-dependent relationship between avoidance and the misinformation effect has previously been reported, further research is needed.

Participants who were more distressed by the film were better able to remember the source of misinformation than less distressed participants. Encouraging source monitoring has previously been found to reduce misinformation errors for trauma analogue material, although higher distress may still engender misinformation endorsement (Nahleen et al., 2022). Interestingly, intrusive symptoms were associated with a smaller misinformation effect during immediate and delayed retrieval. During delayed retrieval, the misinformation effect was almost negligible for participants with higher intrusions. This finding is unexpected. Combining literature on traumatic memory (Brewin et al., 2012; Giosan et al., 2009) and the source monitoring framework (Lindsay & Johnson, 1989; Lindsay et al., 2004), one would expect intrusions to lead to increased source confusion. It is possible that the perceptual details in the misinformation narrative were sufficient to trigger intrusive symptoms (Ehler & Clark, 2000) that led attention away from incoming information and consequently reduced misinformation acceptance. There is evidence that memory for intruding scenes of an analogue trauma film is better preserved over time, compared to memory for non-intruding scenes (Herz et al., 2020). Additionally, perhaps intrusions only engender source confusion in clinical samples, such as in Brewin (2012) who found intrusions (i.e. flashbacks) to be associated with greater source memory errors in a sample of men with post-traumatic stress disorder. Our findings should be interpreted with caution. Our sample showed a tendency to attribute details to ‘both’ sources and were unlikely to endorse details to ‘video only,’ even when this was the correct answer (see Table 2). It is possible that low accuracy on misled items may reflect a tendency to pick the safer, incorrect option (i.e. ‘both’). It is also possible that our source memory findings were affected by order effects, as this task was placed at the end of both phases. Further replication is needed to delineate the source of these results.

We found hyperarousal symptoms to be associated with a smaller misinformation effect on all three memory tasks. This is consistent with a recent review finding that moderate levels of stress and arousal may reduce misinformation when retrieval is delayed (Sharma et al., 2022). Thus, this conclusion may extend to hyperarousal as a symptom of distress. Certain conclusions are limited as this dimension of distress has not always been included in past trauma analogue studies (e.g. Monds, 2016; Nahleen et al., 2020). Our findings indicate its relevance and future studies should also explore this variable.

Our results may be affected by some limitations. We shared the misinformation narrative via audio, which may have led to difficulties comprehending the narrative. While we tried to safeguard against technical or comprehension issues via our exclusion criteria, some participants could have circumvented this by not endorsing technical difficulties and proceeded with the study. However, this appears unlikely given we found a significant misinformation effect. Additionally, the design of the study prevents conclusions on causality to be made. Given our focus on the influence of post-traumatic distress and its symptoms, we did not measure other individual differences that may underlie some of the observed results. For example, overgeneral memory may play a role in the relationship between avoidance and the misinformation effect (Hallford et al., 2019; Williams et al., 2007). It is likely that some participants in our study engaged in emotion regulation strategies besides avoidance to manage their distress in response to the film. Strategies such as trait acceptance following a trauma analogue film have been associated with greater memory recall (Byrne & Kangas, 2022) and may partly explain differences in the misinformation effect found across distress levels. Results may also be affected by presence of psychopathology within our sample, such as post-traumatic stress disorder, which has been associated with higher rates of spontaneous memory distortions (see Otgaar et al., 2017). Inclusion of these trait and psychopathology measures may provide important insight into the relationship between distress and misinformation in future studies. Additionally, although measured twice, the distress levels measured in our study were about a single event and cannot capture the intra-individual differences that may occur in the relationship between distress and misinformation across various events. It will be interesting for future studies to investigate changes in this relationship for each participant when multiple events of varying characteristics are observed.

## Conclusions

To conclude, this study investigated the impact of distress reactions on the misinformation effect across three different memory tasks. Participants reporting higher levels of distress about the film showed a smaller misinformation effect. Memory for consistent details (namely, details that individuals are reminded about) were especially vulnerable to the negative impact of distress, while susceptibility to misinformation was not as impacted and, in some cases, greater distress was associated with reduced susceptibility to misinformation. If results are replicated, there are potential practical implications for legal and clinical contexts in which questioning occurs similarly to in our study. In situations where social influences on memory are of concern, these results indicate that while more distressed witnesses of an event may endorse greater inaccuracies of the event, their accounts may not be as affected by misinformation. Our findings also add to emerging evidence that the short-term use of avoidance may reduce vulnerability to incoming misinformation, while longer-term use increases susceptibility. Given the limited studies in this area, further replication is necessary before conclusions on the effect of long-term avoidance, intrusions, and hyperarousal on suggestion-induced memory distortions can be made.

## Supplementary Information

Supplementary Material 1

## Data Availability

The authors confirm they have reported all measures, conditions, data exclusions, and how sample size was determined. All original study materials, data, and R code are available on Open Science Framework: https://osf.io/6xvzn/?view_only=505386c567be4626a6fbfe8c13aff3c9.

## Abbreviations

PANAS: Positive and negative affect schedule
IES-R: Impact of events scale-revised
Glmms: Generalised linear mixed models
OR: Odds ratios

## Footnotes

1 The misinformation paradigm was first run with a pilot sample of 20 participants. Piloting included only the immediate retrieval (with cued recall and recognition tests only) to ensure the workability of the misinformation paradigm.

2 Note that this film has been used previously by multiple studies (e.g. Strange & Takarangi, 2012) but the details included in the final film vary across studies. For example, the present study did not include clips of the female driver being airlifted to the hospital.

3 Pilot testing revealed respondents frequently confused the identities of the film character referred to in the questions. As such, confusion was avoided by removing some questions (e.g. only the question enquiring about the baby, but not the young child, was retained) and/or adding emphasis to others (e.g. ‘in the first car, how did the woman in the passenger seat die?’). These can be accessed on the study’s OSF entry.
