# Correction: Distress reactions and susceptibility to misinformation for an analogue trauma event

**DOI:** 10.1186/s41235-024-00601-6

**Published:** 2024-10-29

**Authors:** Prerika R. Sharma, Emily R. Spearing, Kimberley A. Wade, Laura Jobson

**Affiliations:** 1 Turner Institute for Brain and Mental Health and School of Psychological Sciences, Monash University, Melbourne, Australia; 2 Law School, University of Exeter, Exeter, UK; 3 Department of Psychology, University of Warwick, Coventry, UK

**Correction: Cognitive Research: Principles and Implications (2024) 9:53** 10.1186/s41235-024-00582-6

The original article erroneously presents incorrectly-swapped labels for the ‘Control’ and ‘Misled’ graphs in Figure 2.

**Fig. 2:**
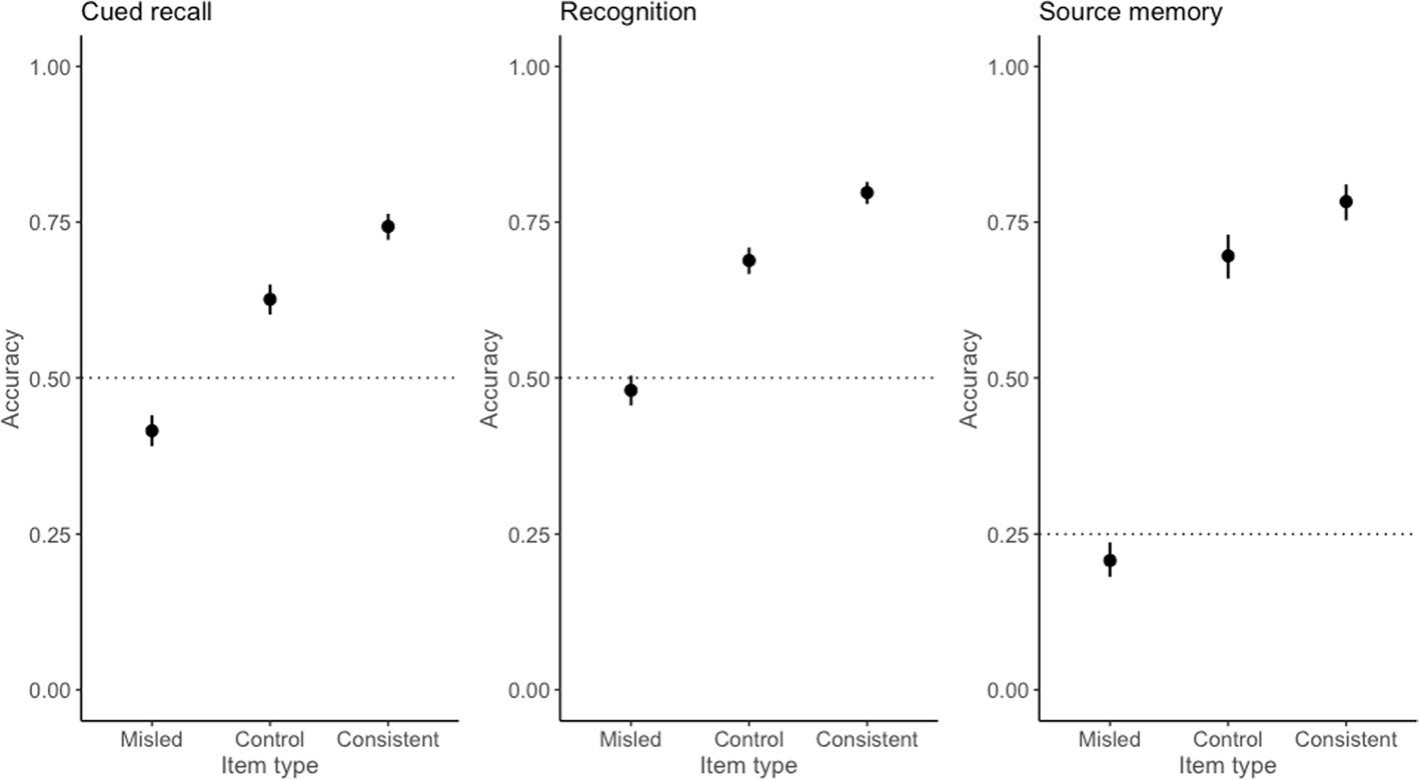
Predicted probabilities of accuracy for misled items for cued recall, recognition, and source memory. *Note*. Dashed line indicates chance performance on each test

The corrected version of Figure 2 can be viewed ahead in this Correction article.

