# Supplementary material for: Distress reactions and susceptibility to misinformation for an analogue trauma event
Source: Cogn Res Princ Implic. 2024 Aug 26;9:53. doi: 10.1186/s41235-024-00582-6 (PMC11345351; doi:10.1186/s41235-024-00582-6)
Supplement: Supplementary file 1 — Supplementary Material 1 [file 41235_2024_582_MOESM1_ESM.docx]

SUPPLEMENTARY MATERIAL

Supplementary material A: descriptive statistics for 199 participants who completed the study in full

Supplementary material B: *glmm* output (non-transformed estimates and odds ratios) for each of the selected models discussed in manuscript’s results. These tables present the interactions for the factor ‘Retrieval time’ that were ommitted from the main text for brevity. Random effects for all models are also presented. These tables combined with the respective tables in the manuscript present the full output of the selected models for each analysis.

# Supplementary material A

Descriptive statistics for sample who completed both phases (*n*=199)

| Variable | Mean (*SD*) (unless specified) | Range |
| --- | --- | --- |
| Age (years) | 40.32 (10.93) | 20 – 72 |
| Gender^a^ (*n*) | 99:97:1:1:1 |  |
| Education^b^ (*n*) | 33:32:88:40:3:3 |  |
| Culture^c^ (*n)* | 2:10:15:23:157:1:1 |  |
| Purpose known (*n*) | 12 |  |
| PANAS Positive affect  Before film  After film | 29.85 (9.88)  27.23 (9.77) | 10 – 50  10 – 50 |
| PANAS Negative affect  Before film  After film | 13.23 (6.48)  17.54 (7.84) | 10 – 45  10 – 49 |
| IES-R Total (Phase 1)  Avoidance  Intrusions   Hyperarousal | 3.47 (2.56)  1.16 (0.86)  1.29 (1.07)  1.02 (0.90) | 0 – 10.25  0 – 3.29  0 – 4  0 – 3.60 |
| IES-R Total (Phase 2)  Avoidance   Intrusions  Hyperarousal | 1.82 (2.22)  0.85 (0.86)  0.57( (0.83)  0.40 (0.75) | 0 – 10.56  0 – 3.71  0 – 4  0 – 3.80 |

# Supplementary Material B

Output for generalised linear mixed models reported in the article: interactions involving retrieval time and random effects of models.

**Table 1**

*Misinformation Effect (Hypothesis 1)*

|  | **Cued recall** | **Recognition** | **Source memory** |
| --- | --- | --- | --- |
| **Random Effects** |  |  |  |
| σ^2^ | 3.29 | 3.29 | 3.29 |
| τ_00_ | 0.27 _Participant_ | 0.19 _Participant_ | 1.07 _Participant_ |
| ICC | 0.07 | 0.05 | 0.25 |
| N | 243 _Participant_ | 243 _Participant_ | 242 |
| Observations | 7992 | 7980 | 6616 |
| Marginal R^2^ / Conditional R^2^ | 0.086 / 0.154 | 0.093 / 0.142 | 0.235 / 0.423 |

*Note.* This table corresponds to Table 2 in the manuscript (subheading: Misinformation effect)

**Table 2**

*Effect Of Total Distress, Retrieval Time, And Item Type On Memory Accuracy (Hypothesis 2, 3)*

|  | **Cued Recall** | | | | | | **Recognition** | | | | | | **Source Memory** | | | | | |
| --- | --- | --- | --- | --- | --- | --- | --- | --- | --- | --- | --- | --- | --- | --- | --- | --- | --- | --- |
| Predictors | Estimates [95% CI] | | O.R. [95% CI] | | p | | Estimates [95% CI] | | | O.R. [95% CI] | | p | Estimates [95% CI] | | | O.R. [95% CI] | | p |
| Control x Delayed retrieval | -0.21 [-0.45 – 0.04] | | 0.81 [0.64 – 1.04] | | 0.095 | | -0.36 [-0.60 – -0.12] | | | 0.70 [0.55 – 0.89] | | **0.004** | 0.16 [-0.15 – 0.48] | | | 1.18 [0.86 – 1.61] | | **0.307** |
| Consistent x Delayed retrieval | -0.32 [-0.58 – -0.07] | | 0.73 [0.56 – 0.93] | | **0.013** | | -0.28 [-0.54 – -0.02] | | | 0.76 [0.58 – 0.98] | | **0.037** | 0.23 [-0.10– 0.55] | | | 1.26 [0.91 – 1.74] | | **0.167** |
| Delayed retrieval x IES-R | -0.00 [-0.05 – 0.04] | | 1.00 [0.96 – 1.04] | | 0.955 | | 0.02 [-0.02 – 0.07] | | | 1.02 [0.98 – 1.07] | | 0.301 | 0.01 [-0.05 – 0.07] | | | 1.01 [0.95 – 1.07] | | 0.692 |
| **Random Effects** | | | |  | |  | |  |  | |  | | |  |  | |  | |
| σ^2^ | | 3.29 | | | | | | 3.29 | | | | | | 3.29 | | | | |
| τ_00_ | | 0.24 _Participant_ | | | | | | 0.19 _Participant_ | | | | | | 1.05 | | | | |
| ICC | | 0.07 | | | | | | 0.05 | | | | | | 0.24 | | | | |
| N | | 243 _Participant_ | | | | | | 243 _Participant_ | | | | | | 242 | | | | |
| Observations | | 7992 | | | | | | 7980 | | | | | | 6616 | | | | |
| Marginal R^2^ / Conditional R^2^ | | 0.091 / 0.154 | | | | | | 0.097 / 0.146 | | | | | | 0.250 / 0.431 | | | | |

*Note.* This table corresponds to Table 2 in the manuscript (subheading: Distress and misinformation effect)

**Table 3**

*Effect Of Types of Distress (Avoidance, Intrusions, Hyperarousal), Retrieval Time, And Item Type On Cued Recall Memory Accuracy (Exploratory Analyses)*

|  | **Avoidance** | | | | | | **Intrusions** | | | | | | **Hyperarousal** | | | | | |
| --- | --- | --- | --- | --- | --- | --- | --- | --- | --- | --- | --- | --- | --- | --- | --- | --- | --- | --- |
| *Predictors* | Estimates [95% CI] | | O.R. [95% CI] | | p | | Estimates [95% CI] | | | O.R. [95% CI] | | p | Estimates [95% CI] | | | O.R. [95% CI] | | p |
| Control x Delayed retrieval | -0.22 [-0.46 – 0.01] | | 0.80 [0.63 – 1.01] | | 0.058 | | -0.15 [-0.39 – 0.10] | | | 0.86 [0.68 – 1.10] | | 0.236 | -0.25 [-0.49 – -0.00] | | | 0.78 [0.61 – 1.00] | | **0.048** |
| Consistent x Delayed retrieval | -0.26 [-0.51 – -0.02] | | 0.77 [0.60 – 0.98] | | **0.033** | | -0.26 [-0.51 – -0.00] | | | 0.77 [0.60 – 1.00] | | **0.047** | -0.41 [-0.67 – -0.15] | | | 0.66 [0.51 – 0.86] | | **0.002** |
| Delayed retrieval x Distress type | 0.02 [-0.10 – 0.14] | | 1.02 [0.90 – 1.15] | | 0.784 | | -0.01 [-0.13 – 0.10] | | | 0.99 [0.88 – 1.11] | | 0.822 | -0.01 [-0.14 – 0.12] | | | 0.99 [0.87 – 1.13] | | 0.871 |
| **Random Effects** | | | |  | |  | |  |  | |  | | |  |  | |  | |
| σ^2^ | | 3.29 | | | | | | 3.29 | | | | | | 3.29 | | | | |
| τ_00_ | | 0.25 _Participant_ | | | | | | 0.25 _Participant_ | | | | | | 0.24 _Participant_ | | | | |
| ICC | | 0.07 | | | | | | 0.07 | | | | | | 0.07 | | | | |
| N | | 243 _Participant_ | | | | | | 243 _Participant_ | | | | | | 243 _Participant_ | | | | |
| Observations | | 7992 | | | | | | 7992 | | | | | | 7992 | | | | |
| Marginal R^2^ / Conditional R^2^ | | 0.089 / 0.154 | | | | | | 0.090 / 0.154 | | | | | | 0.092 / 0.155 | | | | |

*Note.* This table corresponds to Table 4 in the manuscript

**Table 4**

*Effect Of Types of Distress (Avoidance, Intrusions, Hyperarousal), Retrieval Time, And Item Type On Recognition Memory Accuracy (Exploratory Analyses)*

|  | **Avoidance** | | | | | **Intrusions** | | | | | | **Hyperarousal** | | | | | |
| --- | --- | --- | --- | --- | --- | --- | --- | --- | --- | --- | --- | --- | --- | --- | --- | --- | --- |
| *Predictors* | Estimates [95% CI] | O.R. [95% CI] | | p | | Estimates [95% CI] | | O.R. [95% CI] | | | p | Estimates [95% CI] | | O.R. [95% CI] | | | p |
| Control x Delayed retrieval | -0.36 [-0.59 – -0.12] | 0.70 [0.55 – 0.88] | | **0.003** | | -0.31 [-0.55 – -0.06] | | 0.74 [0.58 – 0.94] | | | **0.014** | -0.41 [-0.65 – -0.16] | | 0.67 [0.52 – 0.85] | | | **0.001** |
| Consistent x Delayed retrieval | -0.22 [-0.48 – 0.03] | 0.80 [0.62 – 1.03] | | 0.083 | | -0.20 [-0.46 – 0.07] | | 0.82 [0.63 – 1.07] | | | 0.145 | -0.37 [-0.64 – -0.11] | | 0.69 [0.53 – 0.90] | | | **0.006** |
| Delayed retrieval x Distress type | -0.10 [-0.29 – 0.08] | 090 [0.75 – 1.09] | | 0.277 | | 0.07 [-0.04 – 0.19] | | 1.08 [0.96 – 1.21] | | | 0.212 | 0.07 [-0.05 – 0.20] | | 1.08 [0.95 – 1.22] | | | 0.261 |
| **Random Effects** |  | |  | |  | |  | |  |  | | |  | |  |  | |
| σ^2^ | 3.29 | | | | | | 3.29 | | | | | | 3.29 | | | | |
| τ_00_ | 0.19 _Participant_ | | | | | | 0.19 _Participant_ | | | | | | 0.19 _Participant_ | | | | |
| ICC | 0.05 | | | | | | 0.05 | | | | | | 0.05 | | | | |
| N | 243 _Participant_ | | | | | | 243 _Participant_ | | | | | | 243 _Participant_ | | | | |
| Observations | 7980 | | | | | | 7980 | | | | | | 7980 | | | | |
| Marginal R^2^ / Conditional R^2^ | 0.097 / 0.146 | | | | | | 0.096 / 0.145 | | | | | | 0.099 / 0.148 | | | | |

*Note.* This table corresponds to Table 5 in the manuscript

**Table 5**

*Effect Of Types of Distress (Avoidance, Intrusions, Hyperarousal), Retrieval Time, And Item Type On Source Memory Accuracy (Exploratory Analyses)*

|  | **Avoidance** | | | **Intrusions** | | | **Hyperarousal** | | |
| --- | --- | --- | --- | --- | --- | --- | --- | --- | --- |
| *Predictors* | Estimates [95% CI] | O.R. [95% CI] | p | Estimates [95% CI] | O.R. [95% CI] | p | Estimates [95% CI] | O.R. [95% CI] | p |
| Control x Delayed retrieval | 0.59 [0.31 – 0.88] | 1.35 [1.00 – 1.82] | **0.049** | 0.07 [-0.25 – -0.38] | 1.07 [0.78 – 1.46] | **0.684** | 0.19 [-0.13 – 0.50] | 1.20 [0.88 – 1.65] | **0.251** |
| Consistent x Delayed retrieval | 0.74 [0.46 – 1.02] | 1.51 [1.11 – 2.06] | **0.008** | 0.13 [-0.20 – 0.45] | 1.14 [0.82 – 1.57] | **0.442** | 0.22 [-0.11 – 0.55] | 1.24 [0.90 – 1.73] | **0.194** |
| Delayed retrieval x Distress type | 0.12 [-0.02 – 0.26] | 1.02 [0.87 – 1.19] | 0.838 | 0.29 [0.04 – 0.54] | 1.33 [1.04 – 1.72] | **0.025** | 0.03 [-0.15 – 0.20] | 1.03 [0.86 – 1.23] | 0.753 |
| Control x Delayed retrieval x Distress type |  |  |  | -0.28 [-0.60 – 0.04] | 0.76 [0.63 – 0.91] | **0.085** |  |  |  |
| Consistent x Delayed retrieval x Distress type |  |  |  | -0.43[-0.76 -- -0.10] | 0.65 [0.47 – 0.90] | **0.011** |  |  |  |
| **Random Effects** |  |  |  |  |  |  |  |  |  |
| σ^2^ | 3.29 | | | 3.29 | | | 3.29 | | |
| τ_00_ | 1.07 | | | 1.06 | | | 1.03 | | |
| ICC | 0.24 | | | 0.24 | | | 0.24 | | |
| N | 242 | | | 242 | | | 242 | | |
| Observations | 6616 | | | 6616 | | | 6616 | | |
| Marginal R^2^ / Conditional R^2^ | 0.247 / 0.432 | | | 0.251 / 0.434 | | | 0.251 / 0.429 | | |

*Note.* This table corresponds to Table 6 in the manuscript
